# Ligand-Based and Docking-Based Virtual Screening of MDM2 Inhibitors as Potent Anticancer Agents

**DOI:** 10.1155/2021/3195957

**Published:** 2021-08-05

**Authors:** Bing-Hui Li, Jun-Qi Ge, Ya-Li Wang, Li-Jun Wang, Qi Zhang, Cong Bian

**Affiliations:** ^1^School of Pharmacy and Life Sciences, Jiujiang University, Jiujiang 332000, China; ^2^School of Pharmacy, Jiangxi University of Traditional Chinese Medicine, Nanchang 330004, China; ^3^Institute of Medicinal Biotechnology, Chinese Academy of Medical Science and Peking Union Medical College, Beijing 100050, China

## Abstract

A ligand-based and docking-based virtual screening was carried out to identify novel MDM2 inhibitors. A pharmacophore model with four features was used for virtual screening, followed by molecular docking. Seventeen compounds were selected for an *in vitro* MDM2 inhibition assay, and compounds AO-476/43250177, AG-690/37072075, AK-968/15254441, AO-022/43452814, and AF-399/25108021 showed promising MDM2 inhibition activities with *K*_*i*_ values of 9.5, 8.5, 23.4, 3.2, and 23.1 *μ*M, respectively. Four compounds also showed antiproliferative activity, and compound AO-022/43452814 was the most potent hit with IC_50_ values of 19.35, 26.73, 12.63, and 24.14 *μ*M against MCF7 (p53 +/+), MCF7 (p53 -/-), HCT116 (p53 +/+), and HCT116 (p53 -/-) cell lines, respectively. Compound AO-022/43452814 could be used as a scaffold for the development of anticancer agents targeting MDM2.

## 1. Introduction

The tumor suppressor protein p53 plays a critical role in the regulation of cell proliferation by induction of growth arrest or apoptosis [1]. Overexpression of MDM2 has been found in many cancer cells. The regulatory protein MDM2 binds to the p53 transactivation domain to suppress its transcriptional activity [2]. Therefore, the p53-MDM2 interface has emerged as an important target for the development of novel chemotherapeutic agents.

To date, there are over 20 chemotypes that have been reported as MDM2 inhibitors, such as nutlins [3], spirooxindole [4], benzodiazepinedione [5], isoquinolin-1-one [6], chromenotriazolopyrimidines [7], imidazolyl indole [8], piperidine [9], chalcone [10], diketopiperazines [11], morpholinone [12], and pyrrolin-2-one [13]. Several MDM2 inhibitors, including RG7112 [14], RG7388 [15], SAR405838 [16], AMG-232 [17], HDM201 [18], and APG-115 [19] have progressed into clinical trials. However, none of these inhibitors has reached the clinics.

Thus far, drug resistance and toxicity are still the main challenges of MDM2 inhibitors [20]. Therefore, it is necessary to continue to discover novel inhibitors for the development of anticancer drugs. In the present study, novel MDM2 inhibitors were identified among commercially available compounds using a combination of virtual screening and biological tests. A MDM2 pharmacophore model was first established based on common features of known MDM2 inhibitors, and secondly, the virtual screening was carried out through the pharmacophore model and molecular docking. From a list of retrieved compounds, seventeen were selected for enzymatic assays and five were tested for antiproliferative activity *in vitro*.

## 2. Materials and Methods

### 2.1. Pharmacophore Generation and Validation

**Figure 1:**
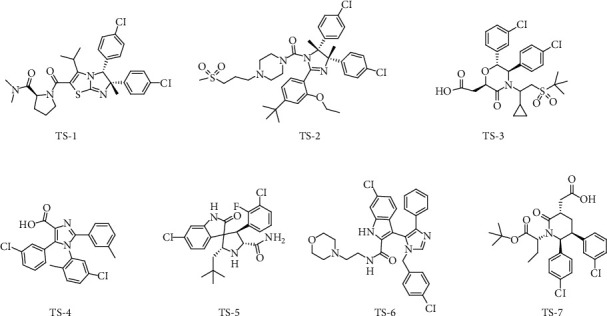
Chemical structures of compounds in the training set.

Common feature pharmacophore models were established using the Discovery Studio 4.5 (DS 4.5, Neo, Trident Technologies LTD) software. Seven MDM2 inhibitors were selected as the training set (Figure 1). All seven ligands were extracted from their crystal structures of MDM2 (Table 1) and checked for bond orders. Hydrogen-bond acceptor (A), hydrogen-bond donor (D), and hydrophobic (H) were specified as the pharmacophore features based on the chemical features of the compounds in the training set. The Principal and MaxOmitFeat values were set to 2 and 0, respectively. The maximum number of hypotheses was set to ten. The maximum feature option was set to 6, and the minimum feature option was set to 4. The minimum distance between features was set to 2.0. All other parameters were at their default.

### 2.2. Pharmacophore Validation

Ten pharmacophore hypotheses were successfully generated. These models were validated according to the rank scores, fit values, experience, and GH score methods. To obtain the fit values, the ligands of the training set were aligned to the ten pharmacophore models using the Ligand Profiler protocol in DS 4.5. Default values were used for all parameters.

The validity of the models was evaluated using the GH score method [21], which is used to evaluate the performance of the pharmacophore model in virtual screening. A decoy set containing 1200 molecules was constructed, among which 50 molecules were known MDM2 inhibitors and the other 1150 molecules were obtained from the Specs database using the Find Similar protocol in DS 4.5. The pharmacophore model was valid with the GH scores ranging from 0.6 to 1. The GH score was calculated by the following formula:

(1) $$\begin{array}{c} \text{GH}=\frac{\text{Ha}\times \left(3\times \text{Ha}+\text{Ht}\right)}{4\times \text{Ht}\times \text{Ta}}\times \left(1-\frac{\text{Ht}-\text{Ha}}{T-\text{Ta}}\right), \end{array}$$

where *T* is the total number of molecules in the decoy set, Ta is the total number of actives in the decoy set, Ht is the number of total hits including actives and decoy molecules, Ha is the number of active hits, and GH is the Güner-Henry score.

### 2.3. Ligand-Based Virtual Screening

The ligand-based virtual screening was performed using the Ligand Profiler protocol in DS 4.5. A database containing 216,347 compounds (from Specs library, http://www.specs.net) was firstly filtered by the “Rule of five.” The “fitting method” was set to “flexible,” and the “maximum omitted features” was set to “-1.” One pharmacophore feature was allowed to be missed when the virtual screening was carried out, and other parameters were set as default. The optimal pharmacophore model (hypo 02) was used as the 3D query. The obtained ligands were then subjected to molecular docking studies.

### 2.4. Docking-Based Virtual Screening

The docking-based virtual screening was carried out using Sybyl-X2.0 (Tri-I Biotech, Shanghai Inc). The crystal structure of MDM2 with PDB code 3LBL was processed through the “Structure Preparation Tool” of Sybyl-X2.0. The ligand and water molecules were firstly removed, and the hydrogen atoms were added. The biopolymer was protonated at pH 7.4, and an Amber7 FF99 force field was applied to it. Other parameters were set by default. The cocrystallization of MDM2 was redocked into the active site of the protein. The RMSD of the cocrystallized and the experimental pose was analyzed, and the value was 0.47 Å, indicating that the protein was suitable for molecular docking. The minimized protein and the compounds selected through the pharmacophore virtual screening were subjected to the docking protocol using the Surflex-Dock Geom mode of Sybyl-X2.0. The pharmacophore establishment, validation, and virtual screening were carried out according to the reported research [22].

### 2.5. MDM2 Binding Assay

The MDM2 binding tests of all 17 compounds were carried out by a fluorescence polarization assay. Compounds were tested at eight concentrations (0.091–200 *μ*M) to obtain *K*_*i*_ values.

30 nM preincubated (30 min) MDM2 and 10 nM PMDM-F peptide in reaction buffer (100 *μ*L, potassium phosphate, 100 mM; bovine gamma globulin, 100 mg/mL; sodium azide, 0.02%) were added into a 96-well plate containing tested compounds at pH 7.5. The plate was incubated at 37°C for 30 minutes, and the polarization values were measured (485 nm excitation, 528 nm static and polarized filter). The *K*_*i*_ values were calculated according to a reported method [23].

### 2.6. Anticancer Activity Test

The anticancer activities of compounds AO-476/43250177, AG-690/37072075, AK-968/15254441, AO-022/43452814, and AF-399/25108021 were evaluated against breast adenocarcinoma cells (MCF7 with wild type p53, p53 +/+), MCF7 (p53 null, p53 -/-), human colon cancer (HCT116 with wild type p53, p53 +/+), and HCT116 (p53 null, p53 -/-) cell lines (ATCC, USA) *in vitro* using the standard MTT assay, with nutlin-3a as the positive control. Compounds were tested at six concentrations (0.001–100 *μ*M).

The cells were seeded into a 96-well plate and incubated at 37°C in 5% CO_2_ for 24 h, after which the test compounds were added to the culture medium and the cells were incubated for 48 h. 20 *μ*L fresh MTT was added to each well, and the cells were incubated at 37°C in 5% CO_2_ for 4 h. After removing the culture medium, 150 *μ*L DMSO was added to dissolve the formazan crystals. The absorbance at 540 nm was measured using a microplate reader. The results, expressed as IC_50_ values, were the average of three determinations and were calculated using nonlinear regression analysis.

## 3. Results and Discussion

### 3.1. Pharmacophore Modeling and Validation

Ligand-based pharmacophore models were established through DS 4.5 software. Seven structurally diverse MDM2 inhibitors were selected as the training set (Figure 1), and the activities of the inhibitors targeting MDM2 are listed in Table 1.

The pharmacophore model was established based on the common features of the training set compounds. As suggested by the Feature Mapping protocol, hydrogen-bond acceptor (A), hydrogen-bond donor (D), and hydrophobic (H) were specified as the pharmacophore features. The top ten hypotheses were generated to identify the necessary common features to inhibit MDM2. Two groups emerged according to the hierarchical clustering analysis: AHHH and DAHH. Statistical parameters of the generated models are listed in Table S1. Rank scores of these models range from 195.19 to 218.23 kcal·mol^−1^. Direct and partial hit values of “1” and “0” show that the training set ligands are mapped onto all features of the model. The max fit of all hypotheses is 4.

**Figure 2:**
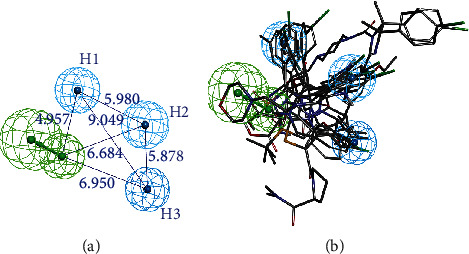
(a) The features of MDM2 pharmacophore model (hypo 02) and distances between the pharmacophore features. The features are colored as follows: hydrogen bond acceptors: green; hydrophobic groups: cyan. The distances are expressed in Å. (b) Compounds of the training set mapped onto hypo 02.

To select the best model from the ten pharmacophore hypotheses, three strategies were employed. (i) There is an unreasonable hypothesis (hypo 03) that the specified chemical characteristics of a small number were deleted. (ii) Four hypotheses (hypo 07, 08, 09, and 10) were not considered because of low rank scores. (iii) The remaining hypotheses were evaluated according to the calculated fit values (Table S2). Hypo 02 and hypo 04 that exhibited high fit values were identified. (iv) Hypo 02 and hypo 04 were further evaluated using the GH scoring method. A database containing 1150 inactive decoy ligands and 50 active MDM2 inhibitors was constructed to evaluate the screening ability of the pharmacophore. The results are demonstrated in Table 2. For hypo 02, among the total hits, 47 active hits were obtained, giving a yield of actives 67.1%. The enrichment factor and GH score were 16.1 and 0.72, respectively. For hypo 04, 42 active hits were obtained, giving a yield of 51.2%. The enrichment factor and GH score were 12.3 and 0.57, respectively. Considering that the hit rate and GH score are the significant parameters in the virtual screening, hypo 02 was chosen as the best pharmacophore model, including one hydrogen bond acceptor group (A) and three hydrophobic groups (H1, H2, and H3) (Figure 2).

### 3.2. Virtual Screening through the Pharmacophore Model and Molecular Docking

**Figure 3:**
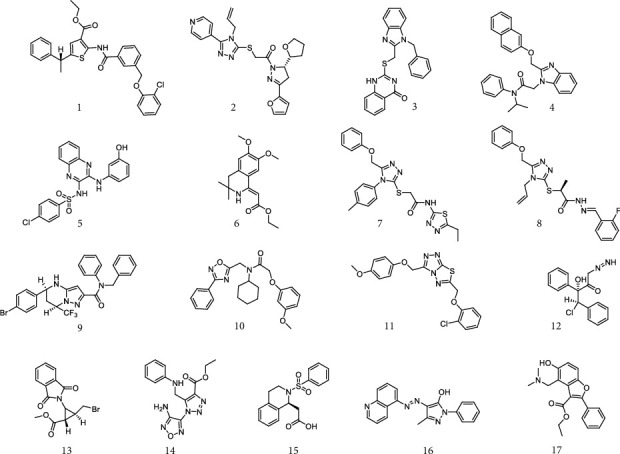
Chemical structures of seventeen compounds identified by virtual screening.

A subset of 216,347 commercially available compounds from Specs was used for virtual screening. “Rule of five” was applied to remove the compounds with unwanted physical and chemical properties, resulting in 147,192 compounds kept in the database. The pharmacophore model was firstly used to screen the database. Based on the fit values (>2), 2936 compounds containing pharmacophore features were identified. The compounds obtained from the pharmacophore matching were docked into the active MDM2. Through molecular docking, 126 compounds with docking scores < −7 kcal mol^−1^ were picked out. The final selection of the screening compounds was according to the docking scores, structural diversity, and interactions between compounds and MDM2. The amino acids surrounding the active site of MDM2 include Met17, Ile19, Val53, Leu54, Phe55, Leu57, Gly58, Gln59, Ile61, Met62, Tyr67, Val75, Leu82, Phe86, Phe91, Val93, Lys94, His96, Ile99, Tyr100, and Ile103 (Figure S2). The compounds which could interact with the amino acids surrounding the active site of MDM2 were chosen. Seventeen compounds were finally selected (Figure 3) and purchased for *in vitro* biological activity. The chemical names and physicochemical properties of the seventeen hit compounds are listed in Tables S3 and S4.

### 3.3. *In Vitro* Enzymatic Assays

The MDM2 binding affinity of all selected compounds was tested, and nutlin-3a was used as a positive control. The results are exhibited in Table 3.

At the initial concentration of 200 *μ*M, eleven compounds showed >50% inhibition (among which compounds AK-968/41172044, AO-476/43250177, AG-690/37072075, AK-968/15254441, AO-022/43452814, and AF-399/25108021 showed >80% inhibition), and the remaining six compounds AG-205/37193004, AK-968/41017877, AO-365/43401788, AB-131/42300827, AD-310/37069010, and AG-690/36631014 showed <50% inhibition. Nutlin-3a exhibited 100% inhibition at 200 *μ*M. Following *K*_*i*_ determinations, compounds AO-476/43250177, AG-690/37072075, AK-968/15254441, AO-022/43452814, and AF-399/25108021 emerged as the potent inhibitors, with *K*_*i*_ values of 9.5, 8.5, 23.4, 3.2, and 23.1 *μ*M, respectively. Compounds AK-968/41172044, AG-690/12071228, AF-399/15336084, AG-690/40754680, AG-205/36869024, and AG-690/36561055exhibited weaker activity, with *K*_*i*_ values in the range of 43.5–126.6 *μ*M. None of the compounds have reported MDM2 inhibitory activities. In addition, seventeen compounds were tested for their Cathepsin K inhibitory activities according to the reported method [22] to evaluate the selective binding of the compounds. The IC_50_ values of all compounds were >200 *μ*M (Table S5). None of the compounds showed Cathepsin K inhibitory activity, indicating good MDM2 binding selectivity.

### 3.4. Interactions between the Compounds and MDM2

**Figure 4:**
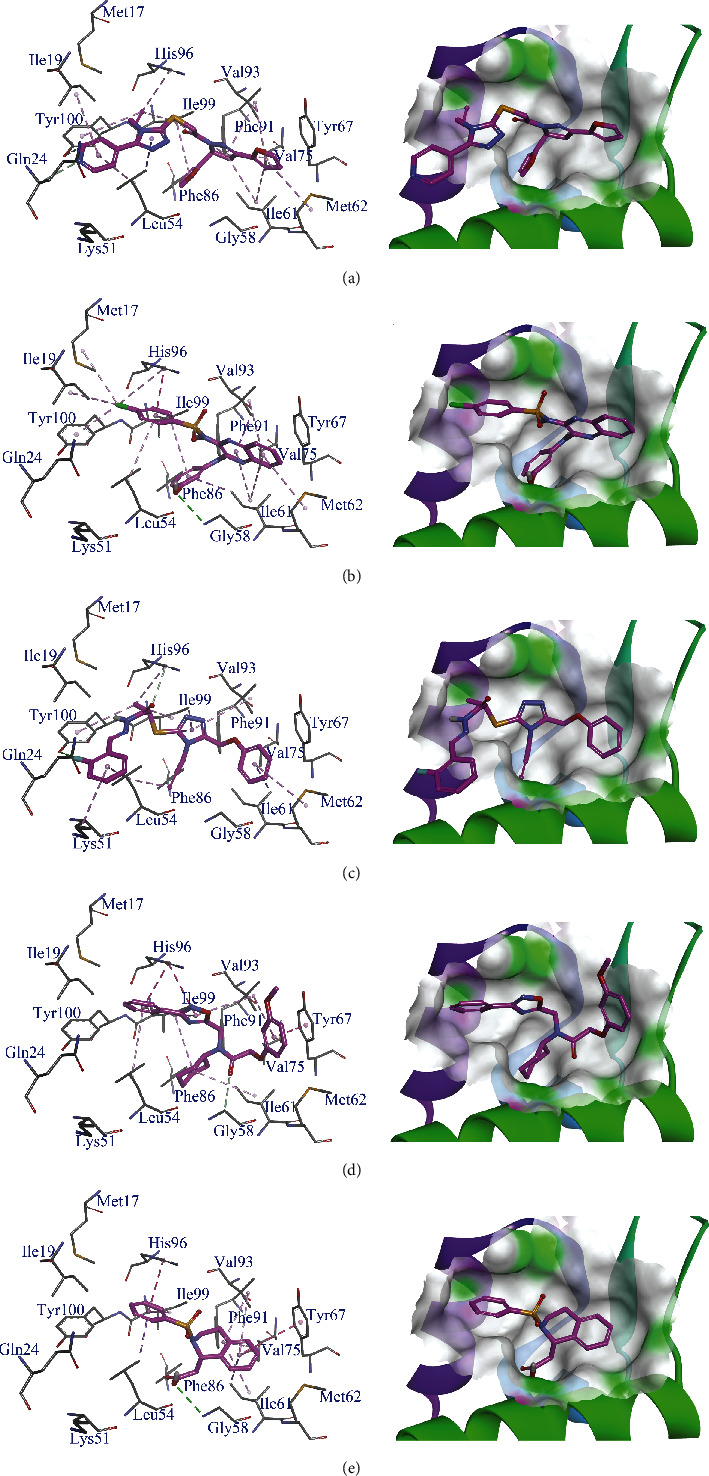
Interactions of compounds AO-476/43250177 (a_1_, a_2_), AG-690/37072075 (b_1_, b_2_), AK-968/15254441 (c_1_, c_2_), AO-022/43452814 (d_1_, d_2_), and AF-399/25108021 (e_1_, e_2_) with MDM2. The amino acids interacting with the compounds are shown in a line form. Hydrogen bonds and hydrophobic interactions are shown in green and pink dashed lines, respectively.

The binding modes of compounds AO-476/43250177, AG-690/37072075, AK-968/15254441, AO-022/43452814, and AF-399/25108021 to MDM2 were analyzed using DS 4.5. The docking results were analyzed to study the possible binding modes of compounds AO-476/43250177, AG-690/37072075, AK-968/15254441, AO-022/43452814, and AF-399/25108021in the active site of MDM2. H-bonds and hydrophobic interactions were observed between the compounds and MDM2. The results are exhibited in Figure 4.

The proposed binding mode of compound AO-476/43250177 is shown in Figures 4(a_1_) and 4(a_2_). Two nonclassical H-bonds and fifteen hydrophobic interactions were observed between compound AO-476/43250177 and MDM2. The nitrogen atom of the pyridine ring and the connected carbon atom serve as an accepter to form nonclassical H-bonds with Gln24 and Tyr100, respectively. The pyridine ring also makes *σ*-*π* hydrophobic interactions with Ile19 and Leu54. The carbon-carbon double bond makes *π*-*π* hydrophobic interactions with Tyr100 and His96 and *σ*-*π* hydrophobic interactions with Ile99. The triazole ring forms a *σ*-*π* hydrophobic interaction with Leu54. The dihydropyridine ring forms *σ*-*π* hydrophobic interactions with Val 75, Val93, and Ile99 and forms *π*-*π* hydrophobic interactions with Phe91. The furan ring connected to the dihydropyridine makes *σ*-*π* hydrophobic interactions with Met62 and Val93, and the other furan ring makes *σ*-*π* hydrophobic interactions with Leu57, Ile99, and Ile103.

The proposed binding mode of compound AG-690/37072075 is shown in Figures 4(b_1_) and 4(b_2_). One classical H-bond and eleven hydrophobic interactions were observed between compound AG-690/37072075 and MDM2. The hydroxyl group donates a classical H-bond to Gly58. The hydroxyl phenyl forms *σ*-*π* hydrophobic interactions with Leu57, Ile61, and Ile99. Chlorophenyl forms *σ*-*π* hydrophobic interactions with Leu54 and Ile99 and forms *π*-*π* hydrophobic interactions with His96. The chlorine atom makes *σ*-*σ* hydrophobic interactions with Met17 and Ile19. The quinoxaline ring makes *σ*-*π* hydrophobic interactions with Ile61, Met62, and Val93.

The proposed binding mode of compound AK-968/15254441 is shown in Figures 4(c_1_) and 4(c_2_). One classical H-bond, one nonclassical H-bond, and eight hydrophobic interactions were observed between compound AK-968/15254441 and MDM2. The fluorine atom acts as a classical H-bond acceptor to Try100. The carbonyl group serves as a H-bond donor to form a nonclassical interaction with His96. The fluorophenyl makes *σ*-*π* hydrophobic interactions with Lys51 and Leu54, and the other phenyl makes a *σ*-*π* hydrophobic interaction with Met62. The methyl forms *σ*-*π* hydrophobic interactions with His96 and Tyr100 and a *σ*-*σ* hydrophobic interaction with Ile99. The triazole ring forms *σ*-*π* hydrophobic interactions with Val93 and Ile99.

The proposed binding mode of compound AO-022/43452814 is shown in Figures 4(d_1_) and 4(d_2_). Two nonclassical H-bonds and ten hydrophobic interactions were observed between compound AO-022/43452814 and MDM2. The carbonyl group serves as a donor to form a nonclassical H-bond interaction with Gly58. The methyl was observed to form a nonclassical H-bond interaction with Tyr67. The phenyl connected to the oxadiazole ring makes *σ*-*π* hydrophobic interactions with Leu54 and Ile99 and a *π*-*π* hydrophobic interaction with His96. The oxadiazole ring forms *σ*-*π* and *π*-*π* hydrophobic interactions with Val93 and His96, respectively. The cyclohexyl ring forms *σ*-*σ* hydrophobic interactions with Leu57, Ile61, and Ile99. The methoxy phenyl forms *π*-*π* and *σ*-*π* hydrophobic interactions with Tyr67 and Val93.

The proposed binding mode of compound AF-399/25108021 is shown in Figures 4(e_1_) and 4(e_2_). One classical H-bond and eight hydrophobic interactions were observed between compound AF-399/25108021 and MDM2. The hydroxyl of carboxyl serves as a donor to form a classical H-bond interaction with Gly58. The phenyl ring connected with sulfonyl forms *σ*-*π* hydrophobic interactions with Leu54 and Ile99 and a *π*-*π* hydrophobic interaction with His96. The other phenyl ring makes *π*-*π* and *σ*-*π* hydrophobic interactions with Tyr67 and Val93, respectively. The piperidine ring makes *σ*-*σ* hydrophobic interactions with Ile61, Val75, and Val93.

### 3.5. *In Vitro* Activity against Tumor Cells

The selected compounds were further tested for their antiproliferative activity in MCF7 (p53 +/+), MCF7 (p53 null, p53 -/-), HCT116 (p53 +/+), and HCT116 (p53 -/-) cells using the MTT assay, and nutlin-3a was used as the positive control.

As the results exhibited in Table 4, compounds AO-476/43250177, AG-690/37072075, AO-022/43452814, and AF-399/25108021 (except compound AK-968/15254441) showed inhibitory activity against cancer cell lines. Compound AO-022/43452814 exhibited more superior activity against all tested cell lines than nutlin-3a. The IC_50_ values of compound AO-022/43452814 were 19.35, 26.73, 12.63, and 24.14 *μ*M against MCF7 (p53 +/+), MCF7 (p53 -/-), HCT116 (p53 +/+), and HCT116 (p53 -/-), respectively. Compound AO-022/43452814 is approximately 20-fold less potent versus MDM2 binding affinity than nutlin-3a but exhibited more potent against cancer cell lines, suggesting that some other nonspecific antiproliferative activities are present. Compound AG-690/37072075 exhibited comparable activity against all tested cell lines against nutlin-3a. The IC_50_ values of compound AG-690/37072075 were 40.17, 56.42, 17.37, and 40.81 *μ*M against MCF7 (p53 +/+), MCF7 (p53 -/-), HCT116 (p53 +/+), and HCT116 (p53 -/-), respectively. Compounds AO-476/43250177 and AF-399/25108021 showed slightly lower activity against HCT116 than nutlin-3a, while no activity (IC_50_ > 100 *μ*M) against MCF7. Consistent with nutlin-3a, compounds AO-476/43250177, AG-690/37072075, AO-022/43452814, and AF-399/25108021 showed greater activity to HCT116 than MCF7. Additionally, the four compounds showed lower IC_50_ values against HCT116 (p53 -/-) and MCF7 (p53 -/-) than HCT116 (p53 +/+) and MCF7 (p53 +/+), demonstrating the selectivity of the compounds over the wild type p53 cancer cell lines. Compound AK-968/15254441 had no or low activity against any of the tested cell lines (IC_50_ > 100 *μ*M). In particular, compound AO-022/43452814 with the most potent MDM2-p53 inhibitory activity (*K*_*i*_ = 3.2 *μ*M) also showed the best antiproliferative activity, and it was chosen for further studies. These compounds have not been reported as anticancer agents.

## 4. Conclusions

In summary, new potent MDM2 inhibitors were identified through the integrative application of virtual screening and biological tests. In this paper, a pharmacophore model was established and successfully used for virtual screening. Five inhibitors (compounds AO-476/43250177, AG-690/37072075, AK-968/15254441, AO-022/43452814, and AF-399/25108021) were identified in enzymatic assays through the biological tests of seventeen hit compounds, with *K*_*i*_ values 9.5, 8.5, 23.4, 3.2, and 23.1 *μ*M, respectively. In the following antiproliferative tests, compounds AO-022/43452814 showed more potent than nutlin-3a, with IC_50_ values of 19.35, 26.73, 12.63, and 24.14 *μ*M against MCF7 (p53 +/+), MCF7 (p53 -/-), HCT116 (p53 +/+), and HCT116 (p53 -/-) cell lines, respectively. Compound AG-690/37072075 showed comparable antiproliferative activity to nutlin-3a. As the same with nutlin-3a, the compounds showed a great difference in activity in the pairing of wild type p53 cells and p53 null cells and more superior activity against wild type p53 cells. In conclusion, we consider that compounds AG-690/37072075 and AO-022/43452814 are promising lead compounds for the development of anticancer drugs targeting p53-MDM2 interactions. Further studies are ongoing to optimize the structure of the compounds and verify the mechanics.

## Figures and Tables

**Table 1:** Activity of compounds in the training set mapped to the pharmacophore model.

Comp.	PDB ID	Potency measure	Potency human MDM2 (nM)
TS-1	3VZV [24]	IC_50_	9.2
TS-2	4IPF [25]	IC_50_	18
TS-3	4OBA [12]	IC_50_	0.4
TS-4	4OQ3 [26]	IC_50_	8
TS-5	3LBL [27]	*K*_*i*_	3
TS-6	4DIJ [8]	IC_50_	30
TS-7	4ERE [28]	IC_50_	4.2

**Table 2:** Statistical parameters and enrichment scores for validation of MDM2 pharmacophore model.

Parameters	Values	
	Hypo 02	Hypo 04
Total molecules in database (*T*)	1200	1200
Total number of actives in database (Ta)	50	50
Total hits (Ht)	70	82
Active hits (Ha)	47	42
% yield of actives [(Ha/Ht) × 100]	67.1%	51.2%
% ratio of actives [(Ha/Ta) × 100]	94.0%	84.0%
Enrichment factor (*E*) [(Ha × T)/(Ht × Ta)]	16.1	12.3
False negatives [Ta-Ha]	3	8
False positives [Ht-Ha]	23	30
GH score	0.72	0.57

**Table 3:** Enzymatic activities of compounds 1-17 identified by virtual screening.

Comp.	Specs ID	Inhibition (200 *μ*M, %)	*K*_*i*_ (*μ*M)	Docking score^a^	Fit value
1	AK-968/41172044	80.2	43.5	–7.34	2.6845
2	AO-476/43250177	95.8	9.5	–7.55	2.7523
3	AG-690/12071228	62.4	103.6	–7.38	2.6610
4	AF-399/15336084	76.3	99.5	–7.32	2.6615
5	AG-690/37072075	90.31	8.5	–7.74	2.7941
6	AG-205/37193004	46.6	>200	–7.43	2.5428
7	AG-690/40754680	63.7	98.3	–7.48	2.6738
8	AK-968/15254441	84.7	23.4	–7.69	2.5464
9	AK-968/41017877	37.6	>200	–7.53	2.5456
10	AO-022/43452814	96.5	3.2	–7.63	2.6915
11	AO-365/43401788	32.3	>200	–7.40	2.6004
12	AB-131/42300827	18.8	>200	–7.17	2.6176
13	AD-310/37069010	42.9	>200	–7.94	2.7319
14	AG-205/36869024	68.6	126.6	–7.06	2.5937
15	AF-399/25108021	82.1	23.1	–7.65	2.7419
16	AG-690/36561055	72.4	64.6	–7.99	2.6924
17	AG-690/36631014	47.3	>200	–7.79	2.7218
Nutlin-3a	—	100	0.15	—	—

^a^Expressed in kcal mol^−1^.

**Table 4:** *In vitro* antiproliferative activities of compounds AO-476/43250177, AG-690/37072075, AK-968/15254441, AO-022/43452814, and AF-399/25108021.

Comp.	Specs ID	IC_50_ (*μ*M) ± SD			
		MCF7 (p53 +/+)	MCF7 (p53 -/-)	HCT116 (p53 +/+)	HCT116 (p53 -/-)
2	AO-476/43250177	>100	>100	35.52 ± 1.48	58.19 ± 4.57
5	AG-690/37072075	40.17 ± 1.77	56.42 ± 4.12	17.37 ± 1.80	40.81 ± 3.54
8	AK-968/15254441	>100	>100	>100	>100
10	AO-022/43452814	19.35 ± 2.26	26.73 ± 1.65	12.63 ± 2.32	24.14 ± 2.31
15	AF-399/25108021	>100	>100	28.12 ± 2.53	51.69 ± 4.15
Nutlin-3a	—	32.18 ± 1.49	53.36 ± 2.81	18.13 ± 2.17	40.72 ± 1.47

## Data Availability

All used data is within the paper.

## Supplementary Materials

Supplementary Materials The following are available online. Table S1: summary of the ten pharmacophore hypotheses for MDM2 inhibitors. Table S2: hit values of compounds in the training set mapped to hypo 01-10. Table S3: chemical names of the seventeen hit compounds. Table S4: physicochemical properties of the seventeen hit compounds. Table S5: Cathepsin K activities of the screened seventeen compounds. Figure S1: two-dimensional (2D) mapping of the pharmacophoric features onto the seventeen compounds obtained from virtual screening. Figure S2: the amino acids surrounding the active site of MDM2.
