# Supplementary material for: Ligand-Based and Docking-Based Virtual Screening of MDM2 Inhibitors as Potent Anticancer Agents
Source: Comput Math Methods Med. 2021 Aug 5;2021:3195957. doi: 10.1155/2021/3195957 (PMC8369186; doi:10.1155/2021/3195957)
Supplement: Supplementary Materials — The following are available online. Table S1: summary of the ten pharmacophore hypotheses for MDM2 inhibitors. Table S2: hit values of compounds in the training set mapped to hypo 01-10. Table S3: chemical names of the seventeen hit compounds. Table S4: physicochemical properties of the seventeen hit compounds. Table S5: Cathepsin K activities of the screened seventeen compounds. Figure S1: two-dimensional (2D) mapping of the pharmacophoric features onto the seventeen compounds obtained from virtual screening. Figure S2: the amino acids surrounding the active site of MDM2. [file 3195957.f1.docx]

Table S1: Summary of the ten pharmacophore hypotheses for MDM2 inhibitors.

| Hypo | Features | Rank | Direct hit | Partial hit | Max fit |
| --- | --- | --- | --- | --- | --- |
| 01 | AHHH | 218.23 | 111111111111111111 | 000000000000000000 | 4 |
| 02 | AHHH | 217.84 | 111111111111111111 | 000000000000000000 | 4 |
| 03 | DAHH | 217.05 | 111111111111111111 | 000000000000000000 | 4 |
| 04 | AHHH | 214.13 | 111111111111111111 | 000000000000000000 | 4 |
| 05 | AHHH | 210.93 | 111110111111111111 | 000001000000000000 | 4 |
| 06 | AHHH | 206.69 | 111111101111111111 | 000000010000000000 | 4 |
| 07 | AHHH | 198.42 | 111101111111111111 | 000010000000000000 | 4 |
| 08 | AHHH | 196.61 | 110111111111101111 | 001000000000010000 | 4 |
| 09 | AHHH | 196.27 | 101111111011111111 | 010000000100000000 | 4 |
| 10 | AHHH | 195.19 | 111111101111111111 | 000000010000000000 | 4 |

Table S2: Hit values of compounds in the training set mapped to hypo 01-10.

| Comp. | Hypo-01 | Hypo-02 | Hypo-03 | Hypo-04 | Hypo-05 | Hypo-06 | Hypo-07 | Hypo-08 | Hypo-09 | Hypo-10 |
| --- | --- | --- | --- | --- | --- | --- | --- | --- | --- | --- |
| **TS-1** | 2.541 | 3.525 | 2.001 | 3.400 | 2.412 | 2.279 | 2.609 | 2.229 | 2.297 | 2.215 |
| **TS-2** | 2.057 | 3.428 | 2.539 | 3.231 | 2.901 | 2.700 | 2.058 | 2.044 | 2.912 | 2.859 |
| **TS-3** | 3.098 | 3.693 | 3.504 | 3.614 | 3.195 | 3. 308 | 3.195 | 3.061 | 3.023 | 3.000 |
| **TS-4** | 3.691 | 3.844 | 3.162 | 3.683 | 3.619 | 3.711 | 3.287 | 3.667 | 3.151 | 3.105 |
| **TS-5** | 3.800 | 4 | 3.285 | 3.963 | 3.739 | 3.894 | 3.985 | 3.842 | 3.549 | 3.772 |
| **TS-6** | 2.000 | 3.069 | 2.615 | 3.006 | 2.302 | 2.706 | 2.046 | 1.828 | 2.188 | 2.586 |
| **TS-7** | 1.225 | 3.146 | 1.539 | 3.246 | 1.649 | 2.458 | 1.735 | 0.866 | 2.463 | 1.286 |

Table S3: Chemical names of the seventeen hit compounds

| Comp. | Specs ID | Chemical name |
| --- | --- | --- |
| **1** | AK-968/41172044 | Ethyl 2-({3-[(2-chlorophenoxy)methyl]benzoyl}amino)-5-(1-phenylethyl)-3-thiophenecarboxylate |
| **2** | AO-476/43250177 | 4-allyl-5-(4-pyridinyl)-4H-1,2,4-triazol-3-yl 2-[3,5-di(2-furyl)-4,5-dihydro-1H-pyrazol-1-yl]-2-oxoethyl sulfide |
| **3** | AG-690/12071228 | 2-{[(1-benzyl-1H-benzimidazol-2-yl)methyl]sulfanyl}-4(1H)-quinazolinone |
| **4** | AF-399/15336084 | N-isopropyl-2-{2-[(2-naphthyloxy)methyl]-1H-benzimidazol-1-yl}-N-phenylacetamide |
| **5** | AG-690/37072075 | 4-chloro-N-[3-(3-hydroxyanilino)-2-quinoxalinyl]​benzenesulfonamide |
| **6** | AG-205/37193004 | Ethyl (6,7-dimethoxy-3,3-dimethyl-3,4-dihydro-1(2H)-​isoquinolinylidene)acetate |
| **7** | AG-690/40754680 | N-(5-ethyl-1,3,4-thiadiazol-2-yl)-2-{[4-(4-methylphenyl)-5-(phenoxymethyl)-4H-1,2,4-triazol-3-yl]sulfanyl}acetamide |
| **8** | AK-968/15254441 | 2-{[4-allyl-5-(phenoxymethyl)-4H-1,2,4-triazol-3-yl]sulfanyl}-N'-(2-fluorobenzylidene)propanohydrazide |
| **9** | AK-968/41017877 | N-benzyl-5-(4-bromophenyl)-N-phenyl-7-(trifluoromethyl)-4,5,6,7-tetrahydropyrazolo[1,5-a]pyrimidine-2-carboxamide |
| **10** | AO-022/43452814 | N-cyclohexyl-2-(3-methoxyphenoxy)-N-[(3-phenyl-1,2,4-oxadiazol-5-yl)methyl]acetamide |
| **11** | AO-365/43401788 | 6-[(2-chlorophenoxy)methyl]-3-[(4-methoxyphenoxy)methyl][1,2,4]triazolo[3,4-b][1,3,4]thiadiazole |
| **12** | AB-131/42300827 | 4-chloro-1-diazo-3-hydroxy-3,4-diphenyl-2-butanone |
| **13** | AD-310/37069010 | Methyl 3-(bromomethyl)-1-(1,3-dioxo-1,3-dihydro-2H-​isoindol-2-yl)-2-aziridinecarboxylate |
| **14** | AG-205/36869024 | Ethyl 1-(4-amino-1,2,5-oxadiazol-3-yl)-5-​(anilinomethyl)-1H-1,2,3-triazole-4-carboxylate |
| **15** | AF-399/25108021 | [2-(phenylsulfonyl)-1,2,3,4-tetrahydroisoquinolin-1-​yl]acetic acid |
| **16** | AG-690/36561055 | 3-Methyl-1-phenyl-4-(quinolin-5-yldiazenyl)-1H-​pyrazol-5-ol |
| **17** | AG-690/36631014 | Ethyl 4-[(dimethylamino)​methyl]-5-hydroxy-2-​phenyl-1-benzofuran-3-carboxylate |

Table S4: Physicochemical properties of the seventeen hit compounds.

| Comp. | Specs ID | MW^a^ | Alogp | N_A^b^ | N_D^c^ | N_RB^d^ | PSA^e^ |
| --- | --- | --- | --- | --- | --- | --- | --- |
| **1** | AK-968/41172044 | 366.78 | 7.95 | 4 | 1 | 11 | 92.87 |
| **2** | AO-476/43250177 | 374.41 | 4.03 | 7 | 0 | 9 | 127.85 |
| **3** | AG-690/12071228 | 416.83 | 4.83 | 5 | 1 | 5 | 120.28 |
| **4** | AF-399/15336084 | 461.32 | 6.37 | 4 | 0 | 8 | 47.36 |
| **5** | AG-690/37072075 | 393.45 | 3.47 | 6 | 3 | 5 | 112.59 |
| **6** | AG-205/37193004 | 339.37 | 2.18 | 3 | 1 | 5 | 56.79 |
| **7** | AG-690/40754680 | 305.29 | 3.94 | 7 | 1 | 10 | 148.36 |
| **8** | AK-968/15254441 | 344.37 | 4.23 | 6 | 1 | 11 | 106.7 |
| **9** | AK-968/41017877 | 359.18 | 6.56 | 5 | 1 | 7 | 50.16 |
| **10** | AO-022/43452814 | 375.45 | 4.31 | 3 | 0 | 9 | 77.69 |
| **11** | AO-365/43401788 | 330.18 | 4.24 | 4 | 0 | 7 | 96.06 |
| **12** | AB-131/42300827 | 320.35 | 5.04 | 3 | 1 | 5 | 37.3 |
| **13** | AD-310/37069010 | 202.22 | 1.07 | 6 | 0 | 4 | 66.69 |
| **14** | AG-205/36869024 | 283.11 | 0.66 | 7 | 2 | 7 | 133.98 |
| **15** | AF-399/25108021 | 237.64 | 2.86 | 5 | 1 | 4 | 83.06 |
| **16** | AG-690/36561055 | 296.12 | 4.91 | 5 | 1 | 3 | 75.66 |
| **17** | AG-690/36631014 | 424.89 | 3.9 | 3 | 1 | 6 | 62.91 |

^a^ MW, Molecular Weight.

^b^ N_A, Number of H-bond Acceptors.

^c^ N_D, Number of H-bond Donors.

^d^ N_RB, Number of Rotatable Bonds.

^e^ PSA, Molecular Polar Surface Area.

Table S5: Cathepsin K activities of the screened seventeen compounds

| Comp. | Specs ID | IC_50_  (μM) | Comp. | Specs ID | IC_50_  (μM) |
| --- | --- | --- | --- | --- | --- |
| **1** | AK-968/41172044 | >200 | **10** | AO-022/43452814 | >200 |
| **2** | AO-476/43250177 | >200 | **11** | AO-365/43401788 | >200 |
| **3** | AG-690/12071228 | >200 | **12** | AB-131/42300827 | >200 |
| **4** | AF-399/15336084 | >200 | **13** | AD-310/37069010 | >200 |
| **5** | AG-690/37072075 | >200 | **14** | AG-205/36869024 | >200 |
| **6** | AG-205/37193004 | >200 | **15** | AF-399/25108021 | >200 |
| **7** | AG-690/40754680 | >200 | **16** | AG-690/36561055 | >200 |
| **8** | AK-968/15254441 | >200 | **17** | AG-690/36631014 | >200 |
| **9** | AK-968/41017877 | >200 | Odancatib | - | 0.001 |

**
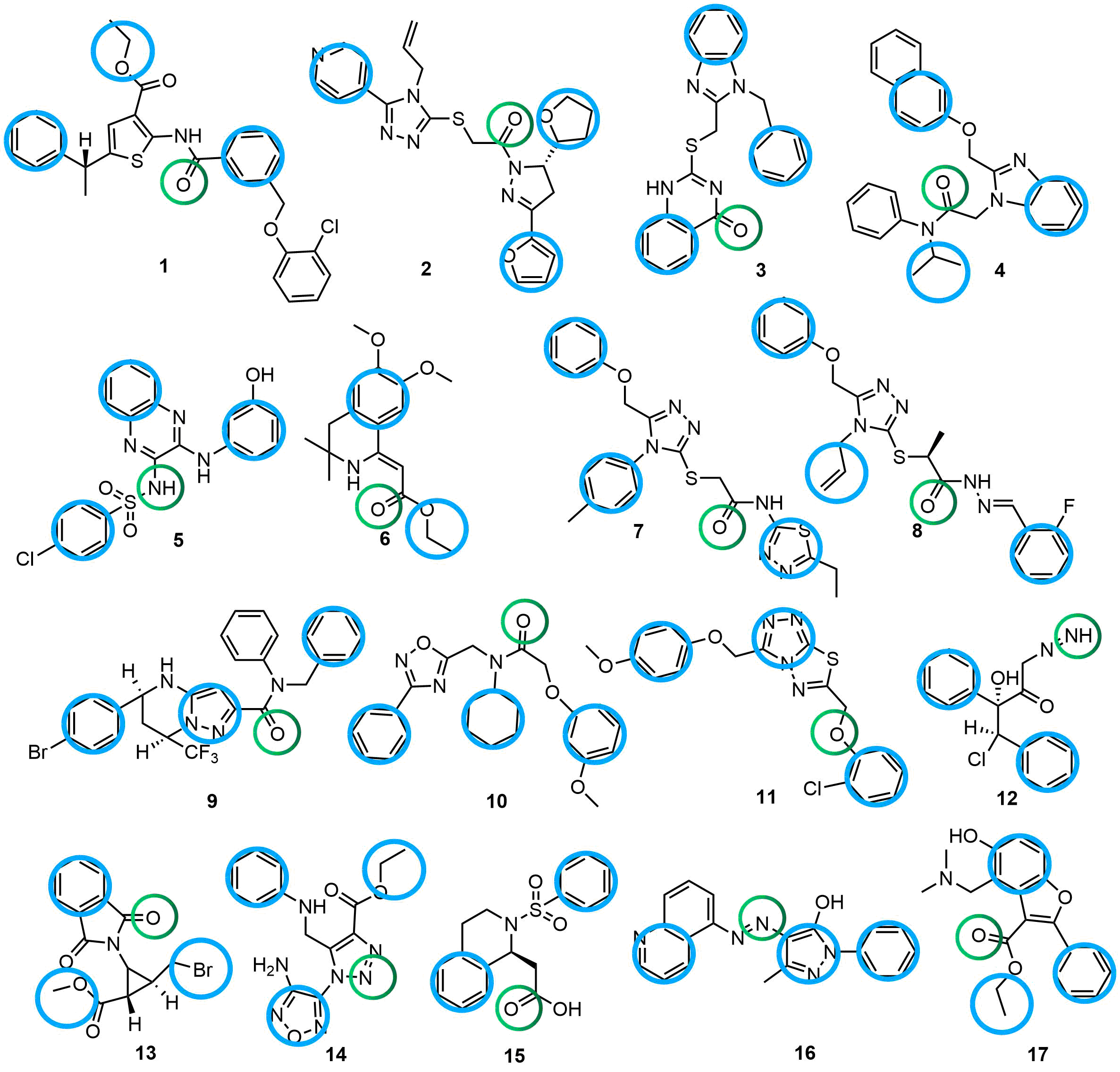
**

Figure S1: Two dimensional (2D) mapping of the pharmacophoric features onto the seventeen compounds obtained from virtual screening.


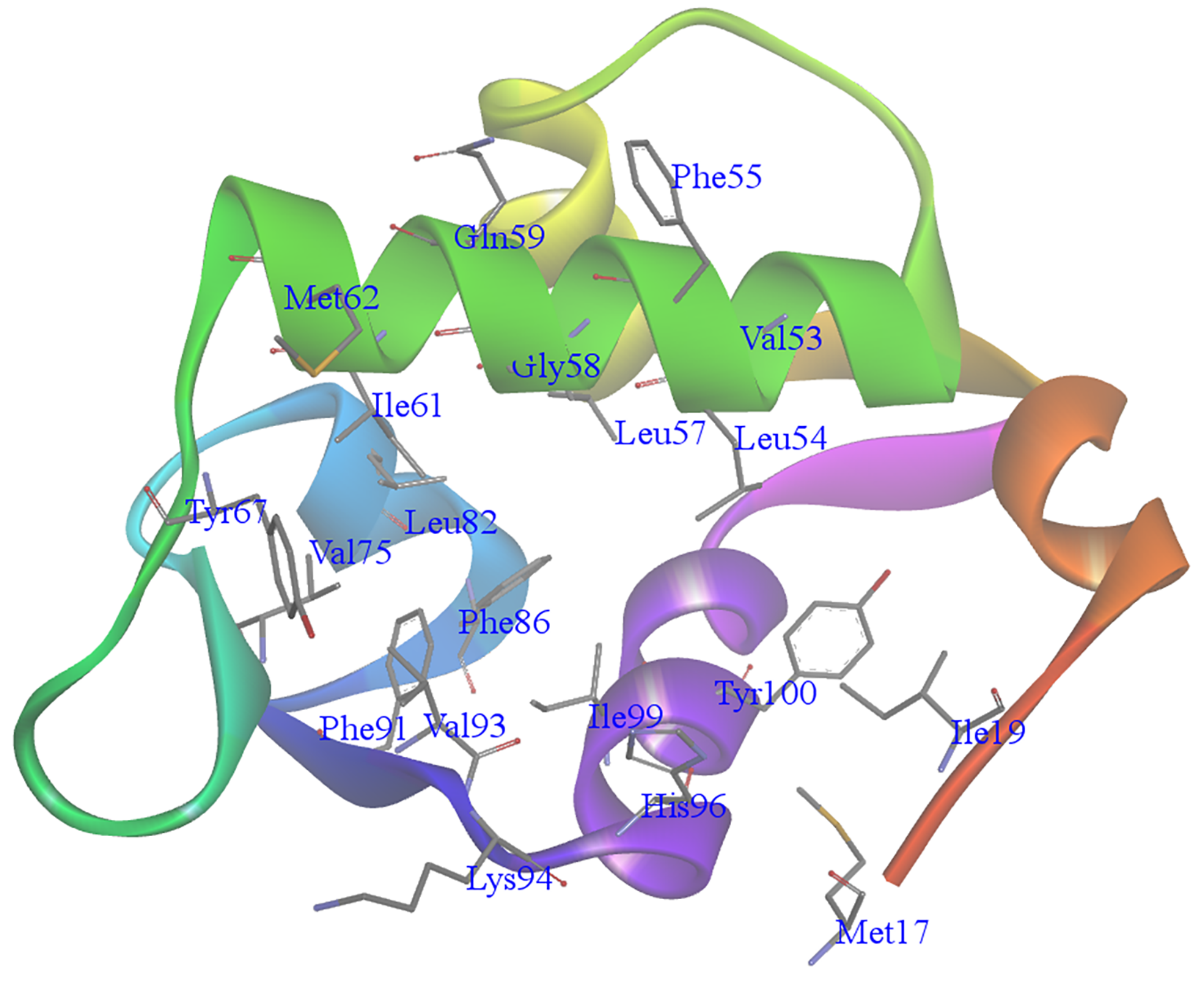


Figure S2: The amino acids surrounding the active site of MDM2.
